# Starting a cultural collective for mothers of children with disabilities: A case study

**DOI:** 10.4102/ajod.v13i0.1367

**Published:** 2024-12-18

**Authors:** Solfrid Raknes, Siv Elin N. Sæbjørnsen, Hege C. Aarlie, Thrine Marie N. Bromstad, Mariana J. Makuu, Caroline Yamala, Sarah Hean

**Affiliations:** 1 Department of Health and Social Care, Molde University College, Molde, Norway; 2 Department of Welfare and Participation, Faculty of Health and Social Sciences, Western Norway University of Applied Science, Bergen, Norway; 3 Department of Sociology and Social work, Faculty of Arts and Social Sciences, The Open University of Tanzania, Dar es Salaam, Tanzania; 4 Uhuru Mama Collective, Dar es Salaam, Tanzania; 5 Department of Social Studies, Faculty of Social Sciences, University of Stavanger, Stavanger, Norway

**Keywords:** Africa, caretakers, children with disabilities, collective, community-based participatory research, peer support, Tanzania

## Abstract

**Background:**

Caring for children with disabilities in Tanzania involves significant challenges, including stigma, limited support and mental health risks. A cultural collective for caretakers of children with disabilities enrolled at a primary school was established to address these issues.

**Objectives:**

The study aims to explore the experiences of caregivers who started a cultural collective and to assess its impact on their lives in the short term.

**Method:**

This study used a community-based participatory research (CBPR) approach with a sequential mixed-methods design. Data were collected over a period of 8 weeks, while the participants in this study established a collective in Dar es Salaam. Quantitative data were analysed using descriptive statistics, and qualitative data were analysed using Braun and Clarke’s method for thematic analysis.

**Results:**

As assessed by a validated and normed questionnaire, Patient Health Questionnaire-9 (PHQ-9), 63% of the caregivers showed signs of depression before starting work in the collective. Economic needs, education and the desire for support were the primary motivations for joining. Starting the collective improved social support, fostered agency and began to enhance caregivers’ financial conditions.

**Conclusion:**

The collective addressed caregivers’ needs for economic improvement, social support and mental support, and the experience was vitalising for the caretakers.

**Contribution:**

This study deepens our understanding of holistic interventions for children with disabilities and their families in urban Africa. It offers valuable insights into a crucial stage of developing contextually relevant interventions for vulnerable, poverty-stricken populations. It provides a model that can be adapted for similar interventions in comparable contexts.

## Background

Caring for a child with special needs can be a highly demanding and an emotionally taxing experience. The caregivers of children with disabilities navigate many cultural, economic, educational and healthcare challenges (United Nations 2019). African caregivers of children with disabilities often lack support structures (Gordon & Bila 2023; Likumbo, De Villiers & Kyriacos 2021; Mkabile et al. 2021). Indigenous African beliefs view disabilities as abnormalities or disruptions in the natural order that require restoration. This condition is typically addressed with the help of religious specialists who attribute it to mystical or supernatural causes such as curses, witchcraft, ancestors or divine intervention (Ndlovu 2016). Hence, caregivers of children with disabilities often face internalised and external societal blame for supposedly bringing misfortune upon their families (Mkabile et al. 2021). Moreover, in the national context of Tanzania, disability still often remains hidden and rarely enters mainstream societal discourse (Aldersey 2012; UNICEF 2023). Not surprisingly, against this backdrop, caregivers of children with disabilities are at an increased risk of mental health problems (Chen et al. 2023), especially in African contexts (Dawkins et al. 2021; Greenwood et al. 2022; Joel et al. 2018; Trafford 2023).

Adequate support systems for caregivers of children with disabilities are essential to securing a brighter future for their children. Research consistently demonstrates that caregivers’ economic stability and mental health are crucial to children’s future outcomes, especially in families dealing with poverty and social challenges (Knifton & Inglis 2020; Wen, Goh & De Mol 2023). Addressing stigma and discrimination by supporting caregivers of children with disabilities is essential for fostering inclusive communities where everyone is valued and respected regardless of ability.

Tanzania ratified the Convention on the Rights of the Child (CRC) in 1991 and enacted the *Law of the Child Act* (2009) to align with its provisions. Furthermore, the country ratified the Convention on the Rights of Persons with Disabilities (CRPD) in 2009, leading to the establishment of the *Persons with Disabilities Act* in 2010. This shows Tanzania’s commitment to advancing the rights of children and persons with disabilities, although challenges persist in full implementation. In addition, Tanzanian policies aim to protect the rights of children with disabilities (e.g., National Strategy for Inclusive Education 2021/22–25/2026, and Guidelines for homeschooling by The Ministry of Education, Science, and Technology 2023), and these have resulted in a significant increase in school enrolment for this vulnerable group. However, a lack of sufficient, accessible and culturally appropriate support structures and interventions means that children with disabilities in Tanzania and their caregivers remain a group at risk of social exclusion, poverty, deprivation and stigma (Knifton & Inglis 2020; Mkabile et al. 2021; Sæbjørnsen, Makuu & Ødegård 2023, Silván-Ferrero et al. 2020; UNICEF Tanzania 2021; UNICEF 2023; World Bank 2022, 2023). The prevalence of disability among children and youth on the mainland of Tanzania is 2.3%, translating to approximately 600 000 individuals. And alarmingly, only 9.4% of families with a member with disabilities are enrolled in any social security schemes (UNICEF 2023).

A review of interventions aiming to support caregivers of children with disabilities suggests that structured parental training programmes can improve parental self-efficacy, especially for those with children under the age of five (Hohlfeld, Harty & Engel 2018). However, none of these studies was conducted in Africa, and the effectiveness of such interventions in the African context, including Tanzania, is yet to be thoroughly examined (Hohlfeld et al. 2018; Kakoko, Kigadye & Hean 2023; McKenzie & McConkey 2016). There is a notable lack of understanding and evidence base that documents the challenges and the characteristics of local and innovative approaches in place to support the caregivers of children with disabilities, especially in an African context. The scarcity of evidence does not mean that no innovations and initiatives are in place in the African context – quite the opposite, as innovation is often stimulated by crisis and need. Low economic resources and a lack of support for children with disabilities and their parents in Tanzania means, therefore, it is ripe for social entrepreneurship and the development of innovative and sustainable alternatives to traditional support models, and research on such initiatives (Connor, Tricia & Bent-Goodley 2016; Teasdale et al. 2023).

In summary, the critical challenges faced by caregivers of children with disabilities include stigma and the attribution of disabilities to supernatural causes, limited access to psychosocial support, insufficient implementation of policies aimed at protecting the rights of children with disabilities and scarcity of evidence-based and innovative approaches tailored the African context to caregivers of children with disabilities. This article aims to provide a deeper understanding of caregivers’ experiences of children with disabilities in Dar es Salaam. It explores the potential of an intervention based on traditional handicrafts and social entrepreneurship to address emotional, social and economic needs.

### The multidimensional impact of collectives

Empowerment is a transformative journey, moving from a state of powerlessness to one of empowerment. Empowerment is a multidimensional concept that involves enhancing individual or collective abilities, rights and capacities to take control of one’s own life and circumstances. Its conceptualisation can vary depending on the context, discipline and theoretical perspective; among these, some are more individual-centric, focusing on the enhancement of women’s abilities and the free exercise of their choices (e.g., Kabeer 1999), and others more community-focused, emphasising group actions and upholding cultural norms that prioritise communal progress (e.g., Budgeon 2015; Kurtiş & Adams 2015). Social cognitive theory highlights the importance of collective efficacy in motivating groups and fostering resilience and performance (Bandura 2000). Collectives have emerged as catalysts for socio-economic and political change for vulnerable populations (Huis et al. 2017; Teasdale et al. 2023) and can have health benefits (Orton et al. 2016). It is crucial to acknowledge the role of collective action facilitated by local social institutions, especially in poverty contexts in East Africa, Andersson and Gabrielsson (2012) argue. Examples of successful East-Africa-based handcraft initiatives that have created better lives are Neema Craft in Tanzania, which employs more than 300 people with disabilities (Neema Craft 2024), and Muya in Ethiopia (Muya 2024).

A ‘collective’ refers to a group formed around a common goal or set of goals, where members work together to achieve outcomes that would be difficult to accomplish individually. Collectives can serve as platforms for mutual support, advocacy, economic cooperation and social change. By blending egalitarianism and feminine expression, contemporary challenges can be addressed (Goss & Heaney 2010). Several models of collectives have evolved:

- *Advocacy Collectives* focus on social change; these groups engage in rights awareness, lobbying and advocacy.
- *Cultural Collectives* challenge societal norms, emphasising cultural preservation through the arts.
- *Self-help Groups* are grassroots entities, typically comprising 10–20 participants, which focus on helping each other through challenges, using mutual savings and borrowing, and accessing more considerable financial opportunities through uniting.
- *Cooperatives* are often larger than self-help groups and involve mutual business ventures, ranging from marketing to production. They are registered and regulated entities under specific acts, making them more structured and legal.
- *Federations* are formed by clustering self-help groups and cooperatives; federations amplify collective influence at broader geographical levels.
- *Enterprise Collectives* are centred on entrepreneurial activities; these groups enable collective production, marketing and sales.

These initiatives can demonstrate, at different levels and forms, models of empowerment that focus on individual beliefs and actions (micro level), relational empowerment (meso level) and societal outcomes as markers of societal empowerment (macro level) (Bronfenbrenner 1981; Huis et al. 2017). A broader literature review (Brody et al. 2015) found that self-help groups could positively impact participants’ economic, social and political empowerment. The timing of outcome assessments and the cultural context in which a study is conducted are crucial factors in evaluating the impact of a collective (Huis et al. 2017). Uhm and Kim (2021) found that among caregivers managing children with special needs, revealing online social support alone had a limited role in caregiving self-efficacy and emphasised the need to prioritise collective empowerment in intervention development to strengthen self-efficacy in this population. Studying disability and inclusion in resource-constrained settings requires integrating local knowledge, like the successful ‘Obuntu Bulamu’ intervention – a peer-to-peer support initiative rooted in local interpretations of belonging and humanity, co-created and successfully tested across ten communities in Central Uganda (Bannink Mbazzi et al. 2020; Nalugya et al. 2023). Likewise, Brogan and Dooley (2024) documented the impact of artisan cooperatives on women in sub-Saharan Africa for dignified and sustainable work to address gender equality and economic growth in the region.

### The Uhuru Mama collective

The Uhuru Mama Collective (UMC) is a cultural collective that aims to empower caregivers of children with disabilities. Learning from the ‘Obuntu Bulamu’ initiative in Uganda (Nalugya et al. 2023), this initiative was grounded in Afrocentric perspectives, a viewpoint that centres on African cultural experiences and knowledge systems, emphasising the importance of African identity, heritage and empowerment (Asante 1991), and Tanzanian values (Mayer, Boness & Louw 2008). The collective comprises 30 caregivers whose children are pupils at a primary school in Dar es Salaam. The school follows an inclusive education model, integrating children with mild disabilities into regular classrooms. Meanwhile, children with more severe disabilities, such as severe intellectual disabilities and blind-deaf children, receive education in specialised classrooms but still share the schoolyard and participate in the broader school culture. Like most public schools in Tanzania, the school struggles with challenges such as overcrowded classrooms, outdated infrastructure, insufficient supplies and a lack of modern technology and specialised staff.

The UMC was established by Dr. Mariana Makuu (the fifth author of this article), a well-connected Tanzanian social worker, and Dr. Solfrid Raknes (the first author of this article), a Norwegian psychologist known for her mental health programmes across economic divides (Raknes 2010a, 2010b, 2014, 2020, 2024). During an academic exchange between Tanzanian and Norwegian educational institutions, they observed that caregivers of children with disabilities were passively waiting in the schoolyard the whole day while their children were educated. Triggered by the observation, they initiated the collective to provide healthy and income-generating activities for the caregivers while their children attended school. Supported by the school’s principal, all caregivers of children with disabilities were invited to an information meeting about starting a cultural collective for caregivers. After a week, an auguration meeting occurred: On 07th July 2023, 30 female caregivers of children with disabilities at the primary school in Dar es Salaam started a cultural collective. They elected a chairperson, an executive secretary and a treasurer to become the leaders of the collective. They formed groups based on product types and began handcrafting items with an initial grant of 540 000 TZH (216 USD). Within 6 weeks, they held their first sales exhibition and received product orders that showed them they could earn money by continuing the collaboration. Dr. Raknes met with the UMC six times during the 8 weeks following the inaugural meeting, guiding the leaders in making informed decisions to create sustainable workplaces for caregivers.

## Aim of the article

This article aimed to explore the perceptions and experiences of caregivers of children with disabilities in Dar es Salaam who joined a cultural collective to gain insight into the potential impact of cultural collectives on marginalised groups in Tanzania. More specifically, the objectives of the article were:

1. To explore caregivers’ experiences of challenges and support as caregivers of children with disabilities in Dar es Salaam.
2. To analyse the caregivers’ experiences of UMC.
3. To examine the short-term impact of participating in UMC on the participants.

## Methodology

### Research design

Rooted in a community-based participatory research (CBPR) framework (Israel et al. 2019; Wallerstein et al. 2018), the study actively engaged caregivers of children with disabilities in planning, data collection and analysis. This collaboration included the researchers who facilitated the collective (first and fifth author), a researcher who is a parent of a child with disabilities herself (second author) and one of the UMC participants (sixth author). The CBPR approach aimed to address power imbalances inherent in traditional research frameworks, as Ocloo et al. (2021) emphasised, and to ensure that the research process was culturally sensitive and contextually relevant, thereby enhancing the depth and applicability of the findings. The study employed a sequential mixed-methods, case study design in line with Yin’s (2018) criteria for addressing ‘how’ and ‘why’ questions. The case study design was chosen specifically to provide an in-depth exploration of the complex, contextual factors affecting caregivers of children with disabilities and the impact of a collective. This approach enabled each phase to serve a distinct purpose, resulting in an integrative study that capitalises on the strengths of both qualitative and quantitative methods (Ivankova, Creswell & Stick 2006).

### Data collection

The study focused on the caretakers’ experiences during the first 8 weeks of the UMC; data were collected in July and August 2023. To facilitate full participation for all participants in the study across literacy levels, assistance was provided by the collective’s secretary (sixth author) and a social worker (fifth author) for completing questionnaires. To maintain confidentiality, names were excluded from the collected questionnaire-based data. A list of names and identity numbers was compiled for internal tracking purposes. For the narratives, the secretary assisted those who wanted help with writing, to write. All data were collected in Swahili, then translated to English by author 6 (the secretary in the collective), and validated by author 5 (researcher and social worker). Trustworthiness, reliability and validity were carefully considered throughout the study to ensure its rigour and credibility, with each aspect systematically addressed to maintain the integrity of the CBPR process and outcomes.

### Sample characteristics

All the 30 caretakers in the collective were invited to participate in the study. They gave informed consent to participate, and 28 completed the questionnaires (*N* = 28). The average age of the participants was 40 years, and all were women. All were caretakers of a child with a disability, and most were the biological mother of that child. The participants reported a daily income for their household of between 0 and 10 000 TZH (equivalent to 3.8 USD). This range reflects the economic reality of many Tanzanian lower-income households. Given Tanzania’s per capita income and the widespread economic challenges, this income range indicates that many of the participants in our sample fall below the poverty line. All were unemployed. At the same time, 59% were their family’s primary earners.

Most participants (68%) had 2–3 children, some had 1 child only and some had 4–5 children. Education varied, with 16 attending primary school for 7 years, six for 4 years, two for 2 years, one for completing secondary school, one for 1 year of university and one never attending school. A total of 64% self-reported their literacy skills in Swahili (which was explained to include writing and reading) to be quite good and 14% reported low Swahili literacy skills. A total of 11% reported having quite good English skills (which was explained to focus on oral communication), while 60% reported having no English skills. The group represented a diverse tribal background. Most lived less than two hours’ travel from the school. A total of 26% of the participants reported not feeling appreciated in the community. Table 1 provides an overview of the demographic and socioeconomic characteristics of the participants in more detail.

**TABLE 1:** Sample characteristics and descriptive statistics.

Participants (*N* = 28)	Mean	*n*	%	Maximum	*n*	%	Minimum	*n*	%	s.d.	*n*	%	Missing data
Age	39.8	-	-	50	-	-	25	-	-	7.14	-	-	1
Mental health (PHQ-9)	7.41	-	-	21	-	-	0	-	-	6.07	-	-	5
**Categorical data**												
Caregivers language skills	Not at all	-	-	A little	-	-	Quite well	-	-	Fluently	-	-	-
Swahili (written)	-	4	14	-	6	21	-	18	64	0	-	-	0
English (oral)	-	17	60	-	8	29	-	3	11	0	-	-	0
Main breadwinner	Yes	16	57	No	12	43	-	-	-	-	-	-	0
Daily income in the household	0	1	4	1000–5000	6	24	6000–10 000	18	72	more than 10 000	-	-	3
Phone access	Smart-phone	8	29	Normal phone	19	68	No phone	1	3	-	-	-	0
Number of children	1	5	18	2–3	18	64	4–5	5	18	6 or more	-	-	0
People in the household	2–3	4	15	4–5	13	50	6–7	5	20	8 or more	4	15	2
Appreciated in community	Yes	16	59	Sometimes	4	15	No	7	26	-	-	-	1

Note: Table 1 provides the demographic attributes of the 28 caregivers who participated in the study. Continuous categories included age and mental health status as assessed by the Patient Health Questionnaire (PHQ-9); categorical variables included caregivers’ literacy skills in English and Swahili, economic role in the family, phone access, number of children, number of people in the household and community appreciation.

s.d., standard deviation; PHQ-9, Patient Health Questionnaire.

### First phase: Questionnaires

The paper-and-pen completed questionnaire-based data were collected during a workday at the collective. The questionnaire designed for this study was structured into seven content areas: Demographics, Family, Economy, Employment, Support, Needs and concerns and Motivations for joining the collective. It concluded with an open-ended segment for additional comments. For more details, see Appendix 1: Questionnaire used for this study. Furthermore, the level of depression was assessed by using a standardised and normed scale validated for use among adults in Tanzania (Fawzi et al. 2019), Patient Health Questionnaire-9 (PHQ-9) (Kroenke, Spitzer & Williams 2001). The PHQ-9 is a nine-item instrument where respondents are asked to indicate how frequently they have been bothered by specific problems over the past two weeks, with answer choices ranging from ‘Not at all’ (scored as 0) to ‘Nearly every day’ (scored as 3). The sum of scores from all nine questions results in a total score ranging from 0 to 27. This total score helps to classify depression severity: 1–4 indicates minimal depression, 5–9 indicates mild depression, 10–14 indicates moderate depression, 15–19 indicates moderately severe depression and a score of 20–27 indicates severe depression.

### Second phase: Narratives, products and observations

This phase consisted of three main components: participants’ narratives, their handcrafted products accompanied by stories they wrote to accompany them and observations by the researchers who met the UMC participants during and directly after the sales exhibition.

#### Narratives

All participants were invited to share stories about themselves and their children, explaining why they joined the collective and their backgrounds, struggles and hopes. Nineteen narratives, ranging from a half-page to three pages, were received and analysed.

#### Handcrafted products and associated stories

The handcrafted products included batik dresses, woven wall decorations, handbags and mats woven in coconut leaves (mkeka). Alongside these handmade products, stories were written by the participants about the products and what they represented.

#### Observations

The psychologist and social worker conducted naturalistic observations of participants during their meetings with the collective and critical activities such as product planning, hand-crafting and sales. This naturalistic observational approach (Mehl & Conner 2012) aimed to capture authentic behaviours, interactions, emotions and insights into the caretakers’ genuine reactions and dynamics. By observing while working together, the researchers could document the participants’ experiences in a real-world context without the influence of structured guides or predefined criteria. This method aligns with central community-based participatory research principles (Wallerstein et al. 2018), emphasising the importance of understanding participants’ lived experiences within their natural environment.

### Data analyses

#### Analysis of quantitative data

Continuous variables’ measures of central tendency (mean) and dispersion (standard deviation, range) were calculated. The PHQ-9 scores were summed and analysed, and descriptive statistics were presented. Frequent distributions and percentages were explored for categorical variables.

#### Analysis of qualitative data

The narratives and the product stories were analysed utilising thematic analysis in line with Braun and Clark’s (2006) six-step method: (1) Familiarising oneself with the data, (2) generating initial codes, (3) searching for themes, (4) reviewing themes, (5) defining and naming themes and (6) writing the article. The first author analysed the qualitative data and performed detailed thematic analyses. This preliminary analysis was then reviewed, refined and enhanced by the second author, who provided unique insights informed by her own experience as a parent of a child with disabilities and an experienced researcher on the theme of disabilities. The fifth author (Tanzanian researcher and social worker) and the sixth author (UMC secretary) then revised and validated the analysis, enhancing the findings’ reliability and cultural sensitivity.

### Ethical considerations

During the study’s planning phase, the three leaders of the UMC – the treasurer, secretary and chairperson – were eager to participate in the research, and their enthusiasm was an initiative to develop this study. After sharing information about basic ethical research principles in studies on vulnerable human beings, ethical considerations were thoroughly discussed with UMC leaders. Participation was voluntary and the participants gave oral informed consent. Withdrawal from the study participation at any time would have had no consequences for any participants. Also, participants were free to skip any uncomfortable questions. The research was conducted as part of a collaboration governed by a Memorandum of Understanding (MOU) between the Ministry of Education, Science and Technology of the government of the United Republic of Tanzania, Molde University College and Western Norway University of Applied Science, valid until 18 November 2027. Specific research clearance for this study was approved under Reference No: MOU 18.11.22–18.11.27.

## Results

### Questionnaire-based results, phase 1, from when they entered Uhuru Mama Collective

#### Caregivers’ motivation to join the collective

Women gave four reasons for joining the collective, listing in decreasing order of frequency their needs and ideas on what is needed for better lives: economics, education and skills, achievement and receiving help. Table 2 gives an overview of these themes and the associated sub-themes of concern, associated citations and how many caregivers reported each type of motivation.

**TABLE 2:** Caregivers’ motivations for joining the collective.

Themes	Frequency
**Capital and economic independence**	16
Capital refers to acquiring capital, growing one’s economic situation and striving for financial independence. Generating income, increasing one’s income and establishing a financial foundation.	
**Education and training**	11
Refers to acquiring education, skills, training and business education – Emphasis on supporting a child’s education, supporting the family financially and establishing a secure future.	
**Achievement and success**	6
Refers to personal success, achievements, becoming a successful entrepreneur and reaching goals.	
**Getting help**	2
Refers to getting help or seeking external assistance.	

Note: Table 2 presents the five reasons why 28 caregivers joined the Uhuru Mama Collective and shows the number of caregivers who reported each motivation.

#### Caregivers’ concerns

Four main themes were revealed, each with its sub-themes: (1) Behavioural and social concerns for the child, (2) burden of care, (3) services for the child in the future and (4) economics (see Table 3 for sub-themes, quotes, and frequency analyses).

**TABLE 3:** Maternal concerns in caring for children with special needs.

Themes/sub-themes	Quotes	Times mentioned
**Behavioural and social concerns for the child**		9
Aggressive behaviour; social safety	‘My concern is when he plays with his friends.’ (ID17, female, caregiver)	
	‘He frequently bites.’ (ID42, female, caregiver)	
	‘As he continues to grow, he becomes very aggressive, he can hurt others.’ (ID13, female, caregiver)	
Discrimination and harassment	‘He is despised and abused.’ (ID41, female, caregiver)	
	‘The child gets harsh words.’ (ID40, female, caregiver)	
	‘My fellow parents who do not have disabled children giving min harsh words.’ (ID19, female, caregiver)	
**Burden of care**		5
Constant care	‘Carry him all the time.’ (ID12, female, caregiver)	
	‘The challenge of looking after him all the time.’ (ID16, female, caregiver)	
	‘Lack of education which will helphim to become self-reliant.’ (ID43, female, caregiver)	
Educational Barriers	‘The challenge I have is to provide him with a good and useful education.’ (ID10, female, caregiver)	
**Services in the future**		4
Parental absence	‘If I die, he will miss all services.’ (ID34, female, caregiver)	
	‘If I leave him or stay away from him.’ (ID25, female, caregiver)	
Future economic and social progress	‘How can he survive economically without me?.’ (ID7, female, caregiver)	
	‘Lack of insurance.’ (ID38, female, caregiver)	
**Economic concerns**		3
Treatment	‘Lack of money for treatment.’ (ID3, female, caregiver)	
Basic needs	‘Necessities like transportation, clothing, and food.’ (ID39, female, caregiver)	
	‘My concern is forward economically and socially, when he grows up and after me and his father.’ (ID7, female, caregiver)	
	‘Economy.’ (ID26, female, caregiver)	

Note: Table 3 gives an overview of the key themes and sub-themes, and example of quotes, coded with the identifiers of the repondents, from the caregivers of children with special needs (*N* = 28) about their worries, and the frequency counts for each theme.

#### Institutional support and communication technology access

When questioned about non-governmental organisation (NGO) support, none of the participants reported receiving any. Most participants used ‘normal’ phones; 1/3 used smartphones and one reported no access to the phone. Most participants with smartphones used multiple social media accounts, including Facebook (FB), Instagram, WhatsApp, TikTok and YouTube. Only a few indicated email usage.

### Depression

**FIGURE 1:**
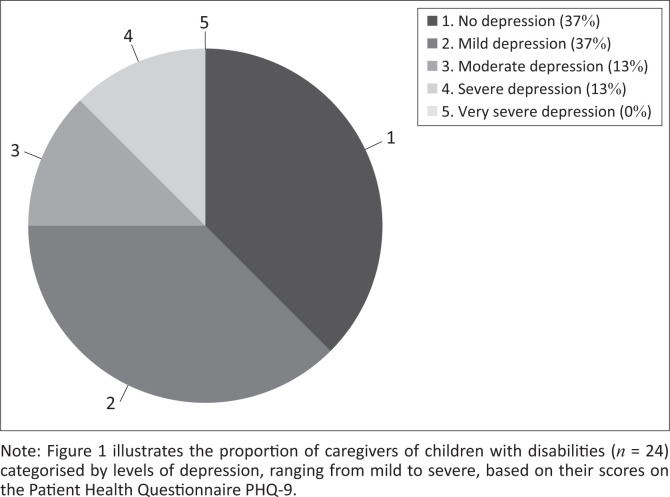
Distribution of depression levels at the beginning of the Uhuru Mama collective.

A total of 62.5% of the participants (*n* = 24) who completed PHQ-9 were found to be struggling with depression. The mean score for depression symptoms was 7.41 (standard deviation [s.d.] = 6.07) (Table 1), significantly elevated compared to average values. As shown in Figure 1, 37.5% showed no sign of depression, 37.5% showed signs of mild depression, 12.5% showed signs of moderate depression and 12.5% showed signs of severe depression, while none showed signs of very severe depression.

### Results based on narratives, handcrafted products and observations, phase 2

#### Narratives

From the narratives about caregivers and their children, six key themes were identified: (1) The child’s Condition, (2) the child’s strengths and struggles, (3) family dynamics, (4) education of the child, (5) financial struggles and (6) future aspirations and community support.

**The child’s condition:** Significant health challenges at birth, including incomplete organs, neonatal pneumonia, jaundice and weight issues, were described in detail. These early health challenges set the stage for continued medical intervention and concerns about developmental milestones. ‘From the minute he was born, we knew we had a long road ahead. The neonatal ICU became our second home’. Most of the narratives indicated some form of developmental delay diagnosed in early childhood, including speech, mobility and cognitive development issues. This led to varying forms of intervention, ranging from physical and speech therapy to specialised medical surgeries, such as eye surgeries for vision impairment. ‘We were so excited for her first steps, but they came much later than expected. It was a celebration but also a reminder’.

**The child’s strengths and struggles:** The caregivers noticed talents in their children, such as good mathematics, music or problem-solving skills. These strengths were focal points for family pride and hope for the future. ‘It is amazing; he can solve complex math problems but cannot tie his shoes. We focus on the math’. Although many children struggled with social interaction because of their conditions, strong bonds were often formed within the immediate family. Caregivers found that these bonds, although fulfilling, sometimes create challenges when the primary caregiver is absent, as others may struggle to understand the child’s unique communication needs. ‘He is not a “social butterfly”, but with us, he is a chatterbox. It is like he speaks his beautiful language’.

**Family dynamics:** Family support was described in many of the narratives: ‘Even on the worst days, her smile lights up the room. And somehow, we get through it as a family’. However, many were single caregivers and had become so after giving birth to a child with disabilities. The child’s father was absent in 15 of the 19 stories, either as not mentioned at all or as mentioned as a father who has left: ‘The challenges I face in raising my child include the fact that his father abandoned us and left the child in my care. I struggle economically to ensure I meet his essential needs in life, including medical care, clothing, bedding, food, and other safety-related care’.

**Education:** Many participants reported experiencing rejection from multiple schools upon disclosing their child’s disability. This resulted in a quest to find the right educational environment for their child, which proved to be an emotional and logistical challenge for the family. ‘We tried three schools in two years. They kept saying they could not meet her “special” needs’.

**Financial struggles:** The financial strain of providing for a child with special needs was a common theme in most narratives. ‘I do not know how we are making ends meet’.

**Future aspirations and community support:** A recurring aspiration among the caregivers was to help their children and other families facing similar challenges. ‘I want to ensure no parent feels as lost as I did’.

### Handcrafted products and associated stories

Each product embodied these caregivers’ challenges, coping strategies and hopes for their children. The products and the stories communicated the caregivers’ difficulties and joys. They offered a multidimensional understanding of what it meant to them to be a mother to a child with a disability, stories of hope, unity and how the collective gives support and new hope (see Table 4).

**TABLE 4:** Artistry and advocacy: Products and their stories.

Product	Story
Batik dresses	‘When I received news about my child’s condition, I suddenly felt like my life had become pitch-dark. I did not know how I would raise this child and how I would live. I could not see how he would live; I lived in darkness. However, as the years went by, I started getting to know my child more, and now his future has been painting my life with many beautiful colours. My child is a beautiful colour in my life’.
Wall decorations	‘Leaves are waste that is not valued when they are cut and dried. Banana leaves are not often used *due to* their brittleness, but when banana leaves are appropriately crafted and designed, we obtain a beautiful and decorative ornament. Similarly, this is the case with our children with special needs. At first glance, they may seem delicate and useless because of their intellectual disabilities. However, they become solid and competent when provided love, cared for, valued, and given opportunities. Let us love them, protect them, and care for them’.
Handbags	‘I am always with you, my child, to help, protect, and defend you against those who mistreat and stigmatise you. You are my dear one, and I will carry you on my shoulders forever to shield you from people with misguided notions about you’.
Tablecloths	‘Giving birth to a child with special needs is not a curse, hex, or sacrifice; instead, it is a blessing just like any other child. These children have love and laughter; they care and feel just like other children. Let us fight misconceptions’.
Floor mats	‘At times, when I reach a point of exhaustion and despair, I decide to sit down. However, when I look at my child and contemplate his tomorrow, I gather the strength to rise and continue to fight for his future. This child is my strength when I am disheartened and weary’.

Note: Table 4 gives an overview of products – batik dresses, wall decorations, handbags, tablecloths and mats – created by caregivers of children with special needs. Each product is accompanied by a narrative illustrating the transformative journey of these caregivers, emphasising themes of resilience, love, advocacy and the redefinition of societal value perceptions.

### Observations

At the establishing meeting, a song came up in the group that all the caregivers sang together while smiling and moving to the rhythm. It was a Swahili activism song and goes like this when translated into English: ‘Mothers are the ones expected to bring about change in their families. We are the ones who can bring about change in our society. Mothers are expected to bring about change in our nation’. The caregivers repeated this song many times and in various settings, with improvised changes in the words used, including fathers, teachers, social workers and researchers. The song usually brought smiles and fostered a strong sense of togetherness.

The products were well-received at the sales exhibition, which made one of the caretakers say, with tears in her eyes: ‘This collective, all of this… It makes me feel like coming up from a deep, dark hole’. The caregivers were happy and proud of their products and the money earned. Altogether, they sold items for 1 605 000 TZH (643 USD) and received a big order that would provide them with another 6 000 000 TZH (2400 USD) if completed. This encouraged the group to continue to meet, learn new skills, make new products for sale and actively start looking for opportunities, raising awareness about children with disabilities through their products and stories. The caregivers seemed vitalised through what they said, the tone in their voices, their body language, how they sang and the energy they put into working at the collective.

## Discussion

This article explored the experiences of caregivers of children with disabilities who started a cultural collective in Dar es Salaam and aimed to gain insight into the potential impact of such collectives in the short term. Several theoretical perspectives resonated with our findings, specifically Hanna Arendt’s concepts of labour, work and action (2018), the Self-Determination Theory (SDT) framework of competence, autonomy and relatedness (Ryan & Deci 2000) and Scarcity Theory (Mullainathan & Shafir, 2013).

### Challenges faced and support needed

The fact that 62.5% struggled with depression at the time they started the collective emphasises the need for mental health interventions. Our findings correspond with previous research showing a heightened prevalence of depression among caregivers of children with disabilities (Chen et al. 2023; Dawkins et al. 2021; Demšar & Bakracevic 2023; Greenwood et al. 2022). These findings underscore the psychological toll of caregiving and highlight the importance of addressing mental health needs within this population.

The caregivers’ reasons for joining the collective were economic, education and skills, achievement and receiving help; these are reasons that reflect the multidimensional needs and aspirations of caregivers caring for children with disabilities, emphasising the importance of holistic support initiatives. The most frequent reason for joining was economic, which aligns with the broader socioeconomic context in Tanzania, where economic opportunities for marginalised groups, such as caregivers of children with disabilities, are often limited (World Bank 2022). The fact that the caregivers focus on immediate financial needs aligns with De Bruijn and Antonides (2022) findings, and the focus on economic independence resonates with the autonomy concept in SDT (Ryan & Deci 2000).

Analysis revealed four main themes of concern among caregivers, including behavioural and social problems, the burden of care, future services for the child and economic worries. Worries like ‘He is bullied and abused’ and ‘He frequently bites’ witness the caregivers’ deep empathy and awareness of the social challenges their children might face and are pained by the thought of their children being hurt, hurting others or being marginalised. Our results align with Mkabile et al. (2021) and Sæbjørnsen et al. (2023), emphasising the societal challenges African caregivers of children with disabilities face. These findings underscore the complex challenges faced by caregivers in caring for children with disabilities and highlight the need for multifaceted support.

The lack of institutional support reported by participants highlights a gap in external assistance for caregivers of children with disabilities within the community. The finding is in line with previous research (Knifton & Inglis 2020; Mkabile et al. 2021; Sæbjørnsen et al. 2023, UNICEF Tanzania 2021; UNICEF 2023; World Bank 2022, 2023). The lack of support underscores the importance of initiatives such as the UMC in providing much-needed support and resources essential to protect children with disabilities and their families. The prevalence of smartphone usage among one-third of the participants suggests an opportunity for leveraging digital platforms to enhance communication and access to support services and, at the same time, raises concerns about digital equity and the potential exclusion of those with limited access to technology in line with findings by Ronda,Vasloo and Grainne (2018). Addressing these disparities will potentially strengthen inclusive participation and support within the collective.

### Love and laborious caregiving

Qualitative analysis of narratives and observations revealed critical themes related to the challenges of caregivers caring for children with disabilities and what UMC meant for them. The handcrafted products created by caregivers served as powerful symbols of resilience, love and advocacy, offering insights into the participants’ lived experiences and aspirations for their children’s future.

The written narratives witnessed caregivers who, despite many challenges, were taking control of their lives, seeking the best for their children and navigating complex sociocultural terrains. Arendt’s notion of labour is evident when the caregivers describe their and their children’s conditions. Labour, as the cyclical process of sustaining life, is reflected in the caregivers’ ongoing efforts to address their children’s health conditions and education. It describes how they support their children to eat, dress and walk and have been carrying them for many years.

Also, the focus on education for their child witnesses focuses on competency, echoing the SDT theory. By emphasising their children’s abilities, caregivers foster a sense of capability and self-worth in their children and themselves as caregivers. Previous research has found that caregivers with scarce financial resources often have concerns about being able to control or defend their mothering (Elliott, Powell & Brenton 2015) and tend to attribute ‘poor’ parenting behaviours to themselves (Cooper 2021). Contrary to this, what is more evident in these caregivers’ narratives is the fighting spirit of the caregivers, the strength of caregivers who advocate for their children’s rights. Despite challenges, caregivers in the collective seem better positioned than those depicted in other African studies (Greenwood et al. 2022; Manono & Claquin-Johnson 2023). In contrast to Likumbo et al. (2021), who described how common it is to hide children with disabilities, these caregivers accompany their children to school and advocate for their rights. The caregivers have likely transitioned from wanting to shield their children with disabilities from the world to their current position of acceptance and advocacy and can find meaning in using their rough experiences to influence and create change among peers and increase awareness about disabilities in the broader community (Szlamka et al. 2022).

### Creating messages arguing for the value of their children

Preparing for and participating in the sales exhibition was a platform for these caregivers to advocate for their children’s rights and values. During the first 2 months of the collective, the caregivers argued that their imperfect children, who may never become independent adults, were still precious, and used their beautiful handcrafted bowls and mats to bring these messages. A mother’s relationship with her child with disabilities and a care-demanding child can be contradictory and complex (Schmidt et al. 2023). However, the caregivers’ demonstrations of love can contribute to a ‘legitimisation’ of love for a child with disabilities so that caregivers who still hide away their child out of fear and shame can also gather the courage to accept, support integration, speak up and fight for their child. Consistent with research highlighting the transformative power of women’s collectives in fostering socio-economic and political change (Huis et al. 2017; Teasdale et al. 2023), the activities of the collective served as powerful tools of empowerment and advocacy. Their communal activities resonate with the community-focused model of empowerment, which emphasises group actions and challenging societal norms (Budgeon 2015; Kurtiş & Adams 2015).

### Caregivers on their way up

The observations made by the social worker and the psychologist witnessed the fact that UMC brought caregivers together around their challenges, the collective helped them to refocus on their skills, made them feel part of a group, less isolated and more everyday related and provided opportunities to develop their skills and competence and constructively use their energy and abilities to earn money. A metaphor used by a mother in the collective, ‘This collective, all of this… It makes me feel like I am coming up from a deep, dark hole’, told about a journey from poverty, pain and ignorance to knowledge, hope and relief. Such a journey can be about a transition from experiencing long-term powerlessness, poverty, being overwhelmed by labour, depression and poor prospects to feeling less hopeless and more confident, using their skills to work, earn some money and increase their economic independence, and participate with their voices and handcrafting skills advocating for change. The transformation resonates with the SDT concepts of autonomy, competence and relatedness and Arendt’s most central concepts.

The transformative spirit of the collective was echoed in the Swahili song the Uhuru Mamas often sang, emphasising the power and expectation to initiate change within their family and society. Singing is expected in a country where propaganda songs have a long tradition and are still active as critical agents for change (Waters & Philhour 2019). The shift of roles from labour to work and action parallels studies in other contexts. The benefits of interventions for parents of children with disabilities of role transition from ‘caregivers’ to ‘educators’ to ‘right defenders’ were recently described in research on interventions for mothers of children with autism in China (Wang et al. 2022). Furthermore, the benefits and importance of participating in the community and using peer support initiatives also resonate with recent research on what is perceived as needed for providing change for children with disabilities in Malawi (Greenwood et al. 2022).

### Short-term impact

The UMC led to a better economy for the participants in the first 2 months of the collective: Normally they did not earn anything while waiting for their children at school, but now they earned a little. Importantly, given the economic challenges associated with living and caring for children with disabilities when living in extreme poverty, initiatives that empower these caregivers economically, such as skill development and micro-financing to start a business, can be crucial for caregivers of children with disabilities and their families. Furthermore, the collective seemed to lead to a sense of more support, agency and increased well-being for the participants, as well as less shame. As stigma and superstitions associated with disabilities remain strong in the Tanzanian society (Mkabile et al. 2021), community-based initiatives to change perceptions about disabilities are needed. Grassroots initiatives, like the UMC, can serve as powerful platforms for caregivers to advocate for their children’s rights and societal acceptance. In Tanzania, where research emerging from local knowledge has substantially developed, it is essential to ensure social work curricula incorporate locally relevant practices, theories and understandings to serve the needs of communities better (Nilsen et al. 2023). Interventions that resonate with local culture and values are likely to be more effective and sustainable than interventions focusing on the individual.

### Limitations and strengths

Small sample size, data collected from caregivers only, context specificity and the short intervention time, are limitations of the study. Furthermore, depression was assessed with a self-completed questionnaire only, and there was no quantitative post-test of depression. The potential for inherent biases, such as confirmation and cultural biases from the researchers, could have influenced the interpretation of data and findings, emphasising the need for caution in generalising the results. Future studies should delve deeper and broaden the research spectrum to validate and expand upon these findings. Longitudinal studies with several assessment points are needed to learn more about the impact of collectives on caregivers of children with disabilities over time.

However, examining the perceptions of caregivers of children with disabilities who had joined a newly created collective designed to support them, using a CBPR approach, ensured that the research was deeply rooted in the lived experiences of the community, enhancing the relevance of the findings. The likelihood of meaningful and actionable outcomes was increased by actively involving community members throughout the research process. The mixed-methods design also provided a comprehensive assessment of the collective’s impact, capturing both measurable changes in financial and psychological well-being and the nuanced personal experiences of the participants, offering valuable insights into understudied populations such as those in urban East Africa.

## Conclusion

This study contributed to international discourses and frameworks around the rights and well-being of children with disabilities and their families. It highlights the urgent need for nuanced, culturally attuned, multi-dimensional interventions in an East African context. Innovation is needed to increase access to relevant and impactful support and services for children with disabilities and their families. By weaving together our findings with previous research and empowerment perspectives, Hannah Arendt’s concepts of labour, work and action, and the Self-Determination Theory, we argue that this study has deepened the insights into the psychological and social intricacies that caregivers of children with disabilities face in Dar es Salaam. Hopefully, this study will stimulate more research on the impact of collectives on caretakers of children with disabilities. In line with previous research, this study highlights the significance of economic empowerment as a means to alleviate stressors (Hohlfeld et al. 2018; Huis et al. 2017). We hope this knowledge informs more impactful, accessible psychosocial interventions to assist caregivers of children with disabilities in Tanzania and comparable settings.

## Appendix 1

### Questionnaire for data collection

#### Theme 1: Demographic information

1. What is your age?
2. Educational background?
3. How well can you read and write Swahili?
  a) Not at all b) a little c) quite well d) fluently
4. How well can you speak English?
  a) Not at all b) a little c) quite well d) fluently
5. What is your tribe?
6. How far away from the school do you live, and in what neighbourhood?

#### Theme 2: Your family

7. Who and how many are living in your household?
8. How many children do you have, and what are their ages?
9. What child(ren) has/has a disability, and what is their disability?
10. What thoughts and feelings did you get when you learned that your child has a disability?
11. How has your family reacted to the fact that you have a child with disability?

#### Theme 3: Economy

12. Are you the primary breadwinner of your family?
  a) Yes b) No
13. Your household income per day?

#### Theme 4: Employment and work-related challenges

14. Are you currently employed, and if so, what type of work do you do?
15. Have you experienced any challenges or changes in your work because of being a mother of a child with a disability?

#### Theme 5: Support systems and services

16. Do you receive any support from government or non-government organisations?
  a) Yes b) No
17. Are there any areas where you feel your child is not receiving adequate support or services?

#### Theme 6: Needs and concerns

18. What kind of support or assistance do you feel would be most beneficial to you and your child?
19. What are your main concerns and challenges in raising your child with disability?

#### Theme 7: Social support and networks

20. Are you connected to any support groups or community organisations related to disability care?
  a) No b) Yes. If yes, how and what is your role there?
21. Do you feel appreciated by your community?
  a) Yes, most of the time b) Sometimes c) No, very rarely.
22. I have a…
  a) Smartphone b) Normal phone c) I don’t have a phone
23. I have an account and can use it on…
  a) Facebook b) Instagram c) TikTok d) WhatsApp e) Mpesa f) Other (specify)…………………

#### Theme 8: Joining this woman’s collective

Complete the following sentence:

For me the most important thing I hope to get from joining this women’s collective is………….

**Anything else you would like to add?**
